# Understanding Autism through the Eyes of Nurses: a Cross‐Sectional Study

**DOI:** 10.1002/brb3.71217

**Published:** 2026-01-19

**Authors:** Monirah Albloushi, Reem Saeed Alghamdi, Mona Alqahtani

**Affiliations:** ^1^ Department of Medical Surgical Nursing, College of Nursing King Saud University Riyadh Saudi Arabia; ^2^ Department of Maternal and Child Healthcare, College of Nursing King Saud University Riyadh Saudi Arabia; ^3^ College of Nursing King Saud University Riyadh Saudi Arabia

**Keywords:** autism spectrum disorder, early detection, misconceptions, nursing knowledge, Saudi Arabia

## Abstract

**Purpose:**

Nurses are key to the early detection of autism spectrum disorder (ASD). However, gaps in the literature and misconceptions can delay care; evidence of this from Saudi Arabia is limited. This study was done to examine nurses’ understanding of autism within the Saudi context, inform targeted educational programs, enhance clinical practice, and contribute to closing the persistent global gaps in nurses’ knowledge of ASD.

**Methods:**

In this study, we employed a cross‐sectional online survey of 180 registered nurses to assess their ASD knowledge and beliefs with the aid of the fourth edition of the Diagnostic and Statistical Manual of Mental Disorders (DSM‐IV‐TR) based criteria and a modified Autism Survey. Moreover, we applied descriptive statistics, chi‐square tests with Cramér's *V*, and Pearson's correlations.

**Findings:**

Most nurses identified core characteristics, such as lack of eye contact (79.7%), social unresponsiveness (79.7%), and interaction difficulties (79.1%); fewer identified symptom onset before 36 months (53.8%). Misconceptions included “cold parenting” (33.7%), association with intellectual disability (26.7%), and belief that ASD can be outgrown (46.1%). The nurses’ endorsement of intervention for speech therapy (96.7%), special education (94.5%), and parental counseling (87.6%) was the highest; no respondent identified the Applied Behavior Analysis. Correlations between demographics and recognition were weak; prior autism education modestly improved the recognition of unusual mannerisms (*r* = 0.155, *p* = 0.037).

**Conclusion:**

Although the awareness of overt ASD traits is high, limited early onset knowledge, persistence, and evidence‐based interventions warrant targeted and culturally informed training.

## Graphical abstract

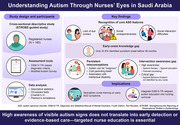

## 1 Introduction

Autism spectrum disorder (ASD) is a neurodevelopmental condition characterized by impairments in social communication and interaction, as well as restricted, repetitive patterns of behavior, interests, or activities (American Psychiatric Association 2013). All racial, ethnic, and socioeconomic groups, as well as individuals from all backgrounds, are affected by ASD (Hodges et al. 2020). Its etiology is multifactorial, involving genetic, biological, and environmental factors (Almandil et al. 2019). The severity of the condition determines the type and intensity of required treatments and interventions needed, since those with more pronounced symptoms may require extensive care and long‐term support (Waizbard‐Bartov et al. 2023).

Globally, ASD affects approximately 1 in every 100 children (Zeidan et al. 2022), with recent US estimates reporting 1 in every 33 children in the United States (Shaw 2025). In Saudi Arabia, prevalence research remains limited; however, emerging studies report a higher regional rate (Al‐Dakroury et al. 2022). For example, AlBatti et al. (2022) estimated a prevalence of 2.51% among children aged 2–4 years, while data from autism centers in Jeddah and Makkah indicated 2.81 per 1000 children (Sabbagh et al. 2021.

Nurses play a vital role in early detection, family education, and multidisciplinary care. However, international research has highlighted considerable variability in nurses’ knowledge and beliefs regarding ASD, which may influence the quality of care delivered. Across diverse settings, nurses have demonstrated familiarity with basic characteristics of autism; however, gaps in competencies related to identification, behavioral interventions, and communication strategies, particularly in culturally diverse or resource‐limited settings, have been reported (Hayat et al. 2019). Analytical synthesis of existing studies shows inadequate recognition of autism features (Govindan and Ramu 2020), moderate awareness of autism's key domains with a significant gap in diagnostic criteria and interventions Ferrara et al. 2024; (Kilicaslan et al. 2024, and very low awareness across all ASD domains, particularly general characteristics, symptoms, diagnostic criteria, and early screening (Ma et al. 2021). These studies show persistent educational and practice‐related gaps across nursing populations.

In Saudi Arabia, research has primarily focused on assessing community awareness rather than nurses’ perceptions and comprehension of ASD (Abdel‐Sattar and El 2022; Abualhommos et al. 2022; AlAlmaei Asiri et al. 2023; Almana et al. 2017; ALRuwaili et al. 2024; Alyami et al. 2022; Mohammed et al. 2024). Studies examining ASD knowledge among nurses remain scarce, limited only to undergraduate students (Alabdulaziz et al. 2024), and pediatric nurses (Alruwaili et al. 2023). Yet, ASD is a lifelong condition, requiring nursing care from nurses across various specialties. This gap highlights the need to evaluate nurses’ broader understanding of ASD within the Saudi context, including their awareness of diagnostic criteria and knowledge of interventions or treatments that can support individuals with ASD. Therefore, this study examined nurses’ understanding of autism within the Saudi context, inform targeted educational programs, enhance clinical practice, and contribute to closing the persistent global gaps in nurses’ knowledge of ASD. More specifically, this study was conducted to: (1) describe nurses’ autism knowledge, perceptions, and diagnostic tendencies; (2) examine differences in agreement with autism‐related statements using chi‐square tests and effect sizes; (3) assess associations between demographic characteristics and diagnostic decisions through Pearson's correlations; and (4) evaluate the strength and practical significance of these associations by reporting p‐values and effect sizes within an exploratory framework.

## 2 Methods

### 2.1 Study Design

This study employed a cross‐sectional descriptive design to assess nurses’ understanding of autism, the diagnostic criteria, and the interventions or treatments required to support individuals with autism. This study was guided by the STROBE observational study checklist.

### 2.2 Population/Sampling

All nurses working in Saudi Arabia were included in the target population. Owing to practical and logistical constraints, recruitment was conducted online through social media platforms and professional networks. This approach constitutes a form of non‐probability convenience sampling, which limited the representativeness of the sample and, consequently, the generalizability of the findings. The inclusion criteria were registered nurses with a minimum of one year of clinical experience working in various hospital departments. The exclusion criteria were nurses with less than one year of clinical experience and individuals from other healthcare professions. The required sample size was calculated using the finite population correction formula for a single proportion, assuming a 95% confidence level, 50% anticipated prevalence, and 7% margin of error for a population of 213,110 nurses in Saudi Arabia (Ministry of Health (MOH) 2023), yielding a minimum of 180 participants (Faul et al. 2009). Finally, 180 registered nurses with different clinical backgrounds participated in this study.

### 2.3 Instrument

The first section inquired about participants’ backgrounds and experiences with autism. The second list included 10 behaviors or characteristics of autism based on the fourth edition of the Diagnostic and Statistical Manual of Mental Disorders (DSM‐IV‐TR) diagnostic criteria. Respondents rated these characteristics as “Necessary,” “Helpful but Not Necessary,” or “Not Helpful” for diagnosing ASD. Among the items in each of the three categories, only the “Necessary” category included factors that are essential for the correct diagnosis of autism. The third section was a modified version of the Autism Survey developed by Stone (1987), which has demonstrated strong psychometric properties (Campbell et al. 1996). These were established during pilot testing of the four‐section questionnaire. Respondents were instructed to rate each statement as “Agree,” “Not Sure,” or “Disagree.” While the DSM‐5‐TR (American Psychiatric Association 2022) is the current standard for diagnosing ASD, the DSM‐IV‐TR criteria were retained to ensure comparability with the validated instrument and earlier studies that assessed nurses’ diagnostic perceptions of ASD. The key diagnostic features relevant to the instrument remained consistent across revisions, minimizing the impact of this choice on construct validity. The DSM‐IV‐TR criteria were used because, at the time of data collection, the validated clinical tools, institutional protocols, and documentation practices in the study setting were still based on DSM‐IV‐TR. Using the existing framework ensured consistency with routine assessment procedures and maintained compatibility with earlier studies conducted in comparable contexts. Furthermore, the diagnostic constructs examined in this research, based on observed symptom patterns and participant narratives, are conceptually stable across DSM‐IV‐TR and DSM‐5‐TR. As reported in prior literature, there is substantial continuity between the two editions for the disorders relevant to this study, suggesting minimal impact on the interpretive validity of findings.

### 2.4 Data Collection

Data were collected using a four‐section, self‐administered online questionnaire distributed via various recruitment methods to ensure completion by nurses working in Saudi Arabia. Participants were recruited online. Online distribution included posting a survey link on Twitter (now X) through professional nursing‐ and healthcare‐related accounts, targeting followers likely to meet the inclusion criteria. A recruitment poster was developed to accompany the online posts. The poster contained a brief description of the study's purpose, inclusion criteria, assurance of confidentiality, and instructions for participation, along with a quick‐response code and shortened hyperlink directing participants to the online questionnaire. To enhance participation rates, the survey link and recruitment poster were shared on three separate occasions spaced approximately 2 weeks apart during the data collection period. Each round of distribution reminded the potential participants of the study's relevance and the voluntary nature of participation. The survey was hosted on Google Forms, and responses were automatically collected and stored in a secure, password‐protected database accessible only to the research team.

### 2.5 Data Analysis

Descriptive statistics (frequencies and percentages) were used to summarize the demographic variables and the distribution of responses. All questionnaire items were completed, yielding a dataset with no missing values. *Chi*‐square tests were conducted to evaluate the statistical significance of nurses’ agreement with autism‐related statements, and all expected cell counts were checked to ensure that they met the chi‐square test assumptions (i.e., no cells with expected counts <5). For each significant *chi*‐square test, Cramér's *V* was calculated to quantify effect size, interpreted using conventional thresholds (small ≈ 0.10, medium ≈ 0.30, large ≥ 0.50). Pearson's correlation analysis was used to assess the relationship between nurses’ demographic characteristics and their diagnostic choices regarding autism‐related traits, with normality and linearity assumptions reviewed for continuous variables.

Given the number of *chi*‐square tests conducted, the results were interpreted with caution regarding the potential inflation of Type I errors. A Bonferroni correction was considered. However, because of the exploratory nature of the study and to avoid an overly conservative adjustment that could mask potentially relevant associations, raw *p*‐values were reported alongside effect sizes to allow readers to make informed judgments. A *p‐*value of <0.05 was considered statistically significant. All analyses were conducted using SPSS version 30.

### 2.6 Ethical Considerations

The study was conducted following the ethical principles outlined in the Declaration of Helsinki. Prior to data collection, ethical approval was obtained from the Institutional Review Board of King Saud University (Approval No. 22–209). All participants received a recruitment statement clearly describing the purpose of the study, procedures, potential risks, anticipated benefits, and measures to protect confidentiality. Participation was voluntary, and individuals were informed that they could withdraw from the study at any time without any consequences. Consent was obtained through the completion and submission of the questionnaire. No personal identifying information was collected, and all responses were anonymized to ensure privacy. The data were stored securely on a password‐protected device accessible only to the research team. In accordance with the approved protocol, all study data will be permanently deleted following the publication of the findings.

## 3 Results

In this study, we employed a structured, four‐part questionnaire designed to assess nurses’ knowledge and perceptions of ASD. The first section gathered participants’ demographic and professional backgrounds. The second section comprised 10 behavioral indicators derived from the DSM‐IV‐TR diagnostic criteria for ASD. Participants rated each item as “Necessary,” “Helpful but Not Necessary,” or “Not Helpful” for diagnosing autism; only items rated “Necessary” were considered essential for accurate diagnosis. The third section comprised a modified version of the Autism Survey developed initially by Stone and Rosenbaum (1988), which demonstrated strong psychometric properties in prior research (Campbell et al. 1996). Respondents indicated their level of agreement with each item on a 3‐point Likert scale: “Agree,” “Not Sure,” or “Disagree.” The final section—not detailed here—addresses intervention‐related perceptions.

### 3.1 Reliability and Internal Consistency

Cronbach's *α* was calculated for two key sections to evaluate the reliability of the instrument. For the DSM‐IV‐TR‐based diagnostic criteria items, *α* = 0.776, and for the modified Autism Survey section, *α* = 0.769, both indicating acceptable internal consistency for early‐stage exploratory research (*α* ≥ 0.70 is generally considered acceptable).

### 3.2 Demographic Characteristics

Of the 180 participating nurses, the majority were female (*n* = 171, 95.0%), aged 35 years and above (*n* = 72, 40%), and had more than 5 years of clinical experience (70.6%). More than half had not previously encountered autism cases in their careers (*n* = 98, 53.8%; Table 1).

**TABLE 1:** Participants’ demographics (*N* = 180).

Variables	Categories	*n*	%
Age	20–25	10	5.6%
	26–30	42	23.3%
	31–35	56	31.1%
	35 and above	72	40.0%
Gender	Male	9	5.0%
	Female	171	95.0%
Duration of clinical experience	0–1	14	7.8%
	1–5	39	21.7%
	5 and more	127	70.6%
In your career, have you encountered any cases of autism?	Yes	84	46.2%
	No	98	53.8%

### 3.3 Nurses’ Perceptions of Autism Spectrum Disorder‐Related Behaviors

As presented in Table 2, nurses overwhelmingly recognized the main characteristics of ASD (social interaction difficulties, lack of eye contact, and lack of social responsiveness) as necessary for diagnosis, a view endorsed by nearly four‐fifths of participants. Language delays, peculiar speech, and rigid or stereotyped play were also widely identified, reflecting an alignment with established diagnostic frameworks. Contrastingly, recognition of “onset before 36 months” was lower (∼54%), with more than one‐third rating it as helpful but not essential, suggesting a potential gap in awareness of age‐of‐onset criteria. Very few respondents considered any characteristic “not helpful,” indicating broad consensus on their clinical relevance, though familiarity with updated DSM guidelines may influence interpretation.

**TABLE 2:** Behaviors/characteristics as suggested by nurses.

Variables	Categories	*n*	%
Language delays	Necessary	130	71.4%
	Helpful but not necessary	45	24.7%
	Not helpful	7	3.8%
Lack of eye contact	Necessary	145	79.7%
	Helpful but not necessary	24	13.2%
	Not helpful	13	7.1%
Need for consistency and resistance to change in routine	Necessary	121	66.5%
	Helpful but not necessary	50	27.5%
	Not helpful	11	6.0%
Peculiar speech characteristics	Necessary	124	68.1%
	Helpful but not necessary	53	29.1%
	Not helpful	5	2.7%
Lack of social responsiveness	Necessary	145	79.7%
	Helpful but not necessary	28	15.4%
	Not helpful	9	4.9%
Rigid or stereotyped play activities	Necessary	133	73.1%
	Helpful but not necessary	42	23.1%
	Not helpful	7	3.8%
Onset of symptoms before 36 months	Necessary	98	53.8%
	Helpful but not necessary	67	36.8%
	Not helpful	17	9.3%
Unusual mannerisms such as finger flicking	Necessary	113	62.1%
	Helpful but not necessary	52	28.6%
	Not helpful	17	9.3%
Preoccupation with objects	Necessary	119	65.4%
	Helpful but not necessary	57	31.3%
	Not helpful	6	3.3%
Social interaction difficulties	Necessary	144	79.1%
	Helpful but not necessary	31	17.0%
	Not helpful	7	3.8%

### 3.4 Suggested Interventions for Autism

Table 3 depicts that nursing professionals overwhelmingly viewed speech therapy (97%) and special education (95%) as necessary interventions for children with autism, indicating a strong awareness of the critical role these services play in supporting communication and developmental outcomes. A significant majority also considered referral to a psychologist essential (85%), reflecting the recognition of the need for behavioral and emotional support. However, a smaller majority (∼68%) viewed referral to a psychiatrist as necessary, with a quarter viewing it as helpful but not necessary. This variation suggests differing perceptions regarding the role of psychiatric interventions in autism care, potentially reflecting differences in clinical exposure or training.

**TABLE 3:** Types of interventions suggested by nursing professionals for autism.

Types	Categories		*n*	%
Speech therapy		Necessary	176	96.7%
		Helpful but not necessary	4	2.2%
		Not helpful	2	1.1%
Special education		Necessary	172	94.5%
		Helpful but not necessary	7	3.8%
		Not helpful	3	1.6%
Referral to a psychiatrist		Necessary	123	67.6%
		Helpful but not necessary	42	23.1%
		Not helpful	17	9.3%
Referral to a psychologist		Necessary	155	85.2%
		Helpful but not necessary	20	11.0%
		Not helpful	1	0.5%

### 3.5 Pharmacological versus Non‐Pharmacological Approaches

**FIGURE 1:**
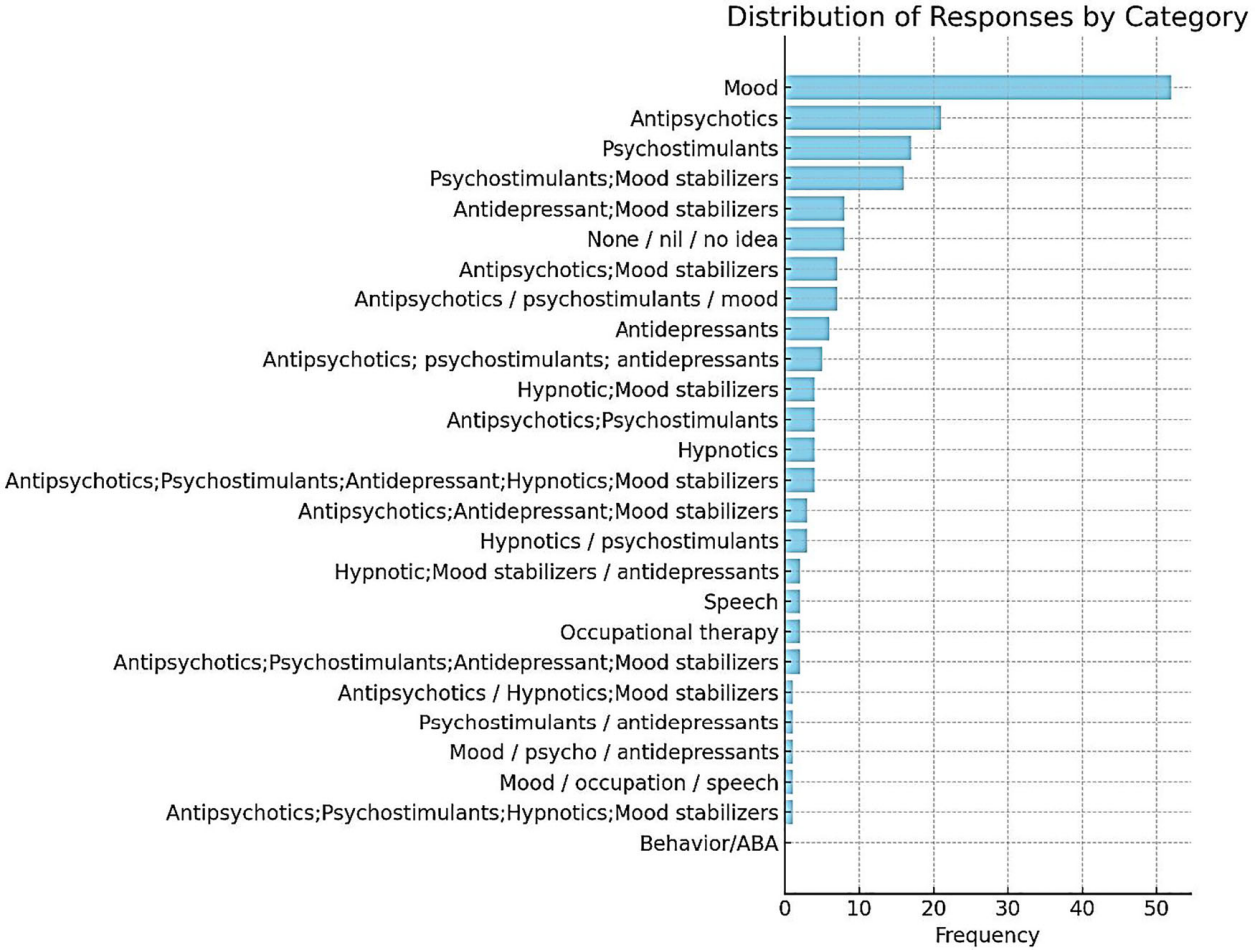
Types of treatment suggested by nursing professionals for autism.

As illustrated in Figure 1, pharmacological interventions were endorsed less frequently than therapeutic or educational approaches. Mood stabilizers (29%) were the most selected medications, followed by psychostimulants (9%) and antipsychotics (12%), with antidepressants and hypnotics rarely cited. No respondents identified Applied Behavior Analysis (ABA) therapy as a treatment option, which may indicate limited awareness of or exposure to this evidence‐based intervention within the clinical context of Saudi nursing practice.

### 3.6 Beliefs and Misconceptions About Autism

**FIGURE 2:**
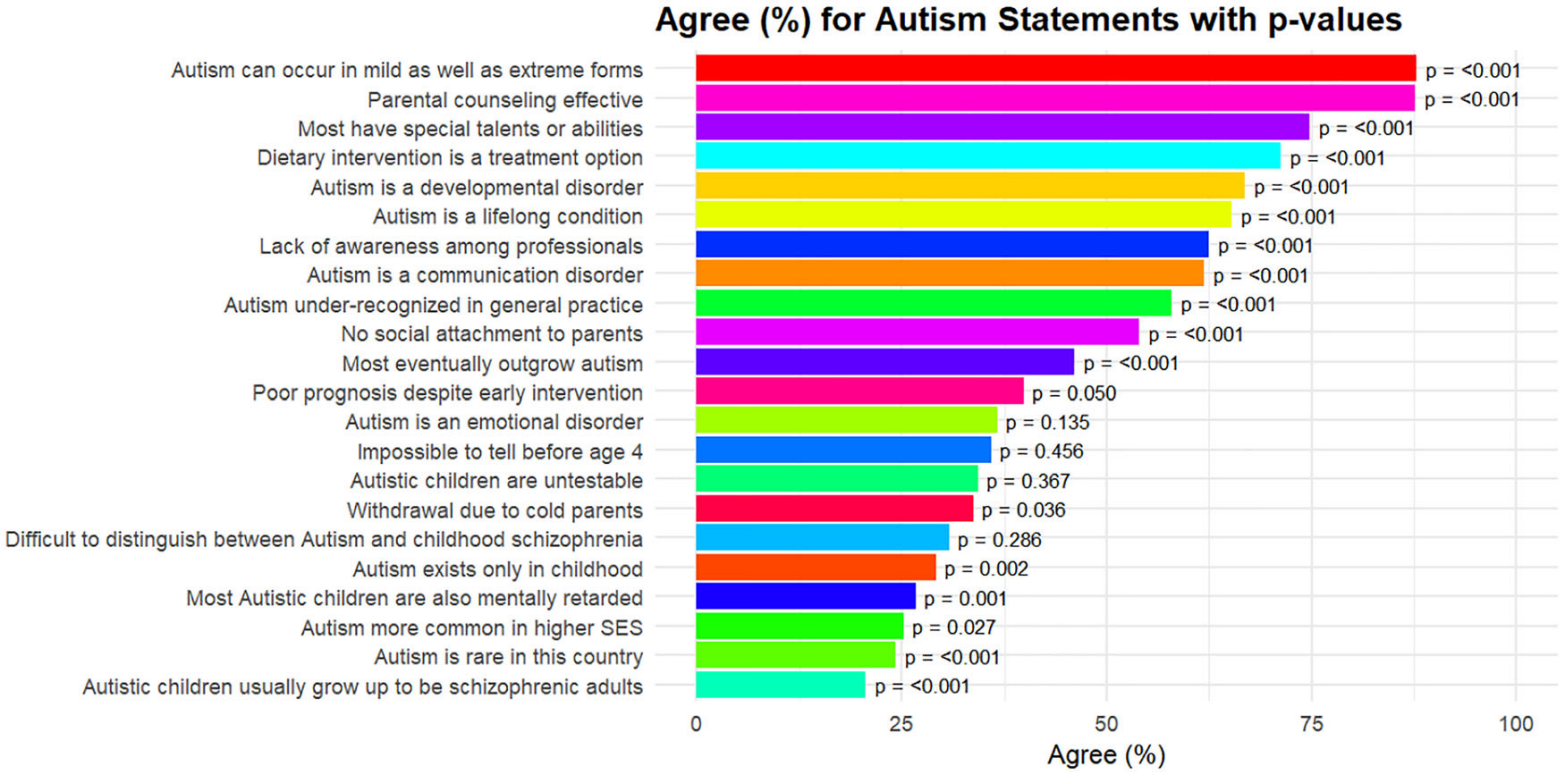
Nurses’ agreement on autism‐related statements with *p*‐value.

As illustrated in Figure 2, most nurses demonstrated a strong conceptual understanding of autism, with the highest agreement occurring in mild and severe forms (87.8%, *V* = 1.15, large), developmental disorders (66.9%, *V* = 0.67, large), and lifelong conditions (65.2%, *V* = 0.59, large). Many were recognized as under‐recognized in practice (57.9%, *V* = 0.48, medium) and as having professional knowledge gaps (62.4%, *V* = 0.55, large). Misconceptions persisted, including beliefs linking autism to “cold parenting” (33.7%, *V* = 0.19, small), higher socioeconomic status (25.3%, *V* = 0.20, small), and intellectual disability (26.7%, *V* = 0.28, small). Ambiguous phrasing reduced the agreement on severity from 87.8% (*V* = 1.15, large) to 20.6% (*V* = 0.50, medium). Most nurses believed that individuals with autism often had special talents (74.7%, *V* = 0.89, large), supported parental counseling (87.6%, *V* = 1.15, large), and endorsed dietary intervention as a treatment option (71.3%, *V* = 0.73, large; Table 4).

**TABLE 4:** Opinion of nursing related to autism statements with a proportion test (*p*‐value).

Opinions	Agree	Disagree	Not sure	Chi‐square with *p*‐value	Cramér's *V*	Effect size interpretation
Autism can occur in mild as well as extreme forms	158 (87.8%)	2 (1.1%)	20 (11.1%)	239.2113 *p* < 0.001***	0.815 (0.75–0.88)	Large
Autistic children usually grow up to be schizophrenic adults	37 (20.6%)	58 (32.2%)	85 (47.2%)	44.855 *p* < 0.001***	0.353 (0.24–0.46)	Medium
Autism is an emotional disorder	66 (36.7%)	51 (28.3%)	63 (35.0%)	4.0876 *p* = 0.135	0.107 (0.00–0.22)	Small
Most autistic children are also mentally retarded	48 (26.7%)	77 (42.8%)	55 (30.6%)	14.034 *p* = 0.001***	0.197 (0.09–0.31)	Small
Difficult to distinguish between autism and childhood schizophrenia	55 (30.9%)	56 (31.5%)	67 (37.6%)	2.578 *p* = 0.286	0.085 (0.00–0.19)	Small
Autism is more common in higher SES	45 (25.3%)	58 (32.6%)	75 (42.1%)	7.2334 *p* = 0.027***	0.143 (0.03–0.26)	Small
Withdrawal due to cold parents	60 (33.7%)	43 (24.2%)	75 (42.1%)	6.6 67 *p* = 0.036	0.137 (0.02–0.25)	Small
Most have special talents or abilities	133 (74.7%)	8 (4.5%)	37 (20.8%)	142.509 *p* < 0.001***	0.670 (0.59–0.74)	Large
Autism is rare in this country	43 (24.2%)	45 (25.3%)	90 (50.6%)	19.678 *p* < 0.001***	0.235 (0.13–0.35)	Medium
Autism is under‐recognized in general practice	103 (57.9%)	23 (12.9%)	52 (29.2%)	40.656 *p* < 0.001***	0.339 (0.23–0.45)	Medium
Lack of awareness among professionals	111 (62.4%)	18 (10.1%)	49 (27.5%)	54.433 *p* < 0.001***	0.392 (0.28–0.50)	Large
Autism is a communication disorder	110 (61.8%)	30 (16.9%)	38 (21.3%)	42.4 *p* < 0.001***	0.346 (0.24–0.46)	Medium
No social attachment to parents	96 (53.9%)	40 (22.5%)	42 (23.6%)	28.24 *p* < 0.001***	0.281 (0.17–0.39)	Medium
Impossible to tell before age 4	64 (36.0%)	62 (34.8%)	52 (29.2%)	1.687, *p* = 0.456	0.097 (0.00–0.21)	Negligible
Autism exists only in childhood	52 (29.2%)	78 (43.8%)	48 (27.0%)	12.676 *p* = 0.002***	0.199 (0.09–0.31)	Small
Poor prognosis despite early intervention	71 (39.9%)	48 (27.0%)	59 (33.1%)	6.01776 *p* = 0.050	0.130 (0.02–0.25)	Small
Autism is a developmental disorder	119 (66.9%)	29 (16.3%)	30 (16.9%)	80.4556 *p* < 0.001***	0.474 (0.37–0.58)	Large
Most eventually outgrow autism	82 (46.1%)	18 (10.1%)	78 (43.8%)	29.0445 *p* < 0.001***	0.286 (0.18–0.40)	Medium
Autism is a lifelong condition	116 (65.2%)	22 (12.4%)	40 (22.5%)	63.4764 *p* < 0.001***	0.421 (0.32–0.53)	Large
Autistic children are untestable	61 (34.3%)	54 (30.3%)	63 (35.4%)	2.0232 *p* = 0.367	0.076 (0.00–0.18)	Small
Parental counseling is effective	156 (87.6%)	1 (0.6%)	21 (11.8%)	237.912 *p* < 0.001***	0.813 (0.74–0.88)	Large
Dietary intervention is a treatment option	127 (71.3%)	15 (8.4%)	36 (20.2%)	94.774 *p* < 0.001***	0.515 (0.42–0.61)	Large

### 3.7 Correlations between Nurse Characteristics and Diagnostic Recognition

**FIGURE 3:**
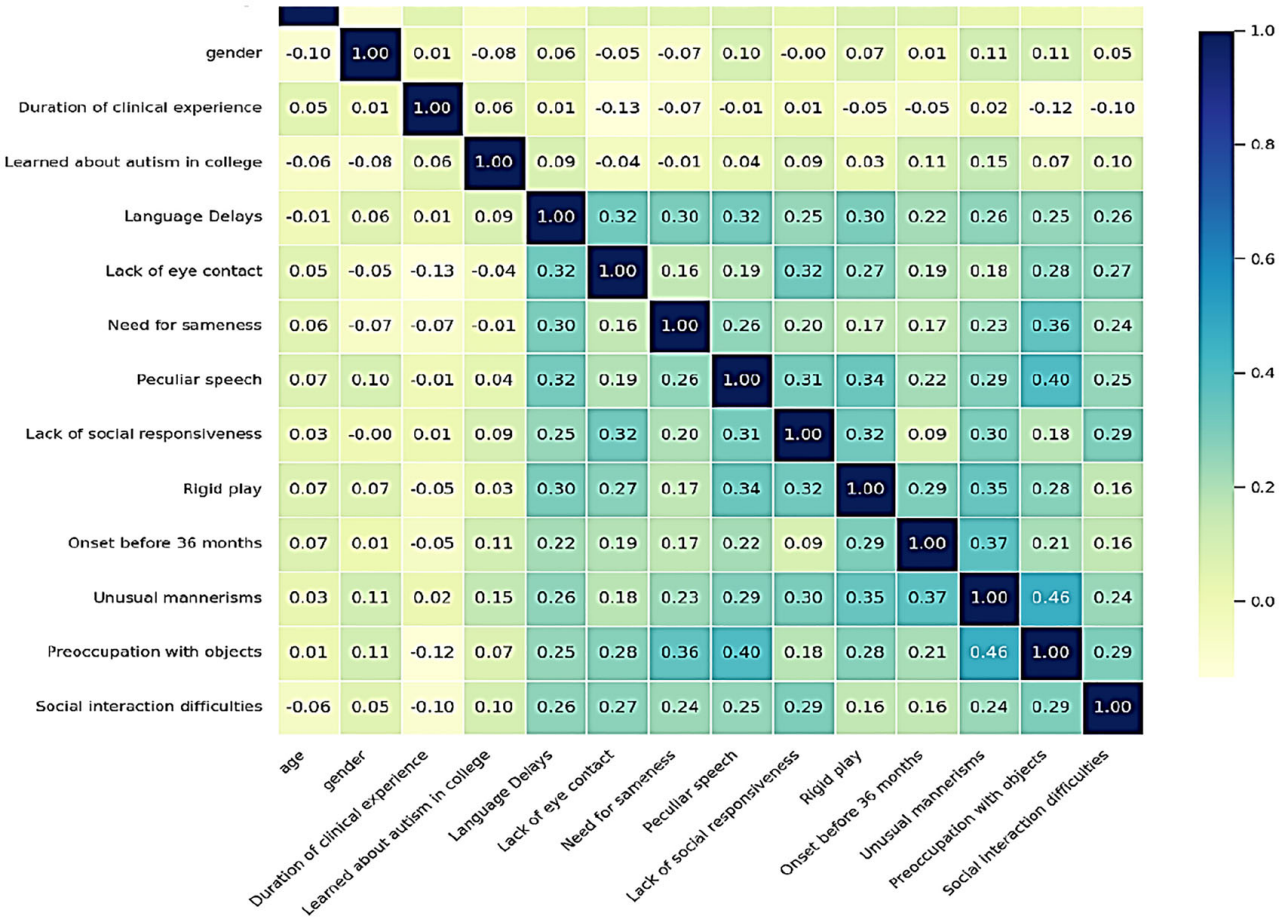
Heatmap between baseline characteristics and suggested autism diagnostics.

Correlations between nurses’ characteristics and recognition of ASD traits were generally weak and non‐significant, with the only notable finding being a modest positive association between formal autism education and the recognition of unusual mannerisms (*r* = 0.155, *p* = 0.037; Figure 3).

## 4 Discussion

This study examined Saudi nurses’ knowledge, perceptions, and diagnostic tendencies regarding ASD and identified key strengths alongside clear areas for improvement. Nurses showed a high ability to recognize core ASD symptoms, such as reduced eye contact, social unresponsiveness, and interaction difficulties, which reflects international trends suggesting that overt social–communication impairments are more readily identified in clinical practice (Dockrell et al. 2019; Hemdi 2020; Petersson‐Bloom and Holmqvist 2022). However, only half of the participants recognized the requirement that symptom onset occur before 36 months (American Psychiatric Association 2022), as specified in DSM‐5‐TR, indicating a critical gap in understanding early developmental indicators. This gap may reflect the extent to which ASD content is embedded within broader nursing curricula and the limited emphasis on early developmental history‐taking markers essential for timely ASD identification (Alenezi et al. 2022). The use of DSM‐IV‐TR–based items may have contributed to some inconsistencies, reinforcing the need for updated educational materials aligned with contemporary diagnostic criteria.

Despite accurate recognition of key symptoms, several misconceptions persisted, including beliefs that ASD is an emotional disorder, is linked to intellectual disability, or is caused by parenting style. Nearly half believed autism could be “outgrown,” highlighting limited awareness of ASD as a lifelong neurodevelopmental condition (Elsabbagh et al. 2012). These misconceptions reflect longstanding international patterns and indicate insufficient autism‐specific training in undergraduate and continuing professional education. They may also influence nurses’ perceived importance of early intervention and timely referral.

Intervention preferences showed strong endorsement of evidence‐based, non‐pharmacological approaches such as speech therapy, special education, and parental counseling, consistent with global best‐practice guidelines with the WHO Caregiver Skills Training Programme (2022) and NICE guidelines (2021). However, psychiatric referral was less frequently selected than psychological referral, despite the high prevalence of comorbid mental health conditions in individuals with ASD (Al‐Beltagi 2021). Mental health stigma and limited integration of psychiatric services into primary care may partially explain this trend within the Saudi context (Dockrell et al. 2019). Such stigma influences referral practices, uptake of interventions, and engagement in mental health services at multiple levels among providers, families, and the broader community (Al‐Beltagi 2021; Papadopoulos et al. 2019; Phelan et al. 2023). Families and caregivers of individuals with autism face significant psychosocial barriers shaped by hopelessness, self‐esteem, and cultural beliefs, which exacerbate their reluctance to seek help and contribute to internalized stigma.

Notably, no participants identified ABA therapy as an intervention, despite its established evidence base. This absence likely reflects limited exposure to behavioral therapies in nursing education and the restricted availability of ABA services in public healthcare settings. The complete lack of ABA therapy selection among respondents is especially concerning, given ABA's strong empirical support as a leading evidence‐based intervention for autism (Smith and Iadarola 2015). This gap may stem from limited exposure to ABA during nursing education or from misconceptions about its role, particularly given that speech therapy and special education predominate in interventions. In Saudi Arabia, ABA services remain less accessible within the public healthcare sector and are generally available only through private or specialized centers, primarily in major cities such as Riyadh, Jeddah, and Dammam (Alasiri et al. 2024; Alenezi et al. 2022). Consequently, nurses in public or decentralized settings might have limited opportunities to observe or refer individuals with ASD to ABA‐based therapies. Reports have identified significant service gaps and limited referral pathways for behavioral interventions, with many families relying on out‐of‐pocket private services (Alenezi et al. 2022). Along with interdisciplinary training emphasizing behavior‐based interventions, integrating ABA education into nursing curricula could address this knowledge gap and promote more comprehensive evidence‐based care for children with ASD in Saudi Arabia.

Diagnostic tendencies did not vary by demographic or clinical experience variables, suggesting that knowledge gaps are broadly distributed across nursing groups rather than concentrated within specific subpopulations (Cashin et al. 2025; Chukwueloka 2016). The modest association between prior ASD education and recognition of unusual mannerisms indicates that brief or embedded exposure to autism content is insufficient. Dedicated ASD modules that use culturally relevant case examples and clinical simulations may support more robust diagnostic knowledge and confidence. This uncertainty echoes prior findings in Saudi Arabia and other low‐ to middle‐income countries (Alsehemi et al. 2017; Chukwueloka 2016). The use of DSM‐IV‐TR‐based items may have influenced participants' responses, particularly regarding behaviors such as age at onset and symptom categorization, which were reconceptualized in DSM‐5‐TR. The transition to dimensional‐ and spectrum‐based criteria in newer editions underscores the need for curricular updates and future studies employing DSM‐5‐TR‐aligned instruments, despite our findings remaining valuable for assessing baseline knowledge (Zwaigenbaum et al. 2013).

Broader system‐level factors may also shape nurses’ knowledge and clinical behavior. The lack of national ASD screening protocols limited public awareness initiatives, and cultural reluctance to accept developmental labels may inhibit early recognition and referral, particularly in rural areas with fewer specialized resources. Because this study was conducted in an urban tertiary hospital, further research is needed to explore how knowledge and attitudes differ across regions and levels of care.

### 4.1 Recommendations and Implications

Three key recommendations emerged to advance ASD care in Saudi Arabia following the frameworks established by the World Health Organization (WHO 2022) and the United Nations (United Nations (UN) 2018). First, it is essential to integrate mandatory, standalone ASD‐ specific educational modules, incorporating the updated DSM‐5‐TR diagnostic criteria (American Psychiatric Association 2022) and culturally relevant case studies into undergraduate and postgraduate nursing curricula. Second, structured, ongoing, and continuing professional development programs focused on early detection and lifelong development of ASD, and comprehensive referral procedures that emphasize the recognition of common psychiatric comorbidities are desperately needed. Third, misconceptions and stigma may be reduced by empowering nurses as leaders in community outreach through MOH initiatives and leveraging the existing maternal and child health infrastructure to broaden public awareness. Embedding these strategies within the Saudi Vision 2030 Health Sector Transformation Program aims to modernize and integrate the healthcare system to improve population health outcomes (MOH 2021), aligning policy imperatives with clinical and educational reforms. To enhance understanding and impact, future research should employ qualitative methodologies to explore referral barriers; utilize direct observational measures of clinical practice; and encompass multidisciplinary, multisite sampling across rural, urban, and primary care settings. It is important to assess changes in knowledge and referral behaviors through a rigorous evaluation of educational interventions using randomized controlled designs. Moreover, it is essential to include the perspectives of families and other healthcare professionals to gain a comprehensive understanding of the challenges in the diagnostic and care pathways in Saudi Arabia.

### 4.2 Strengths and Limitations

The generalizability of the findings to similar clinical contexts is improved due to the use of a validated, psychometrically robust instrument and the inclusion of an experienced, relatively large sample (with approximately 71% having over five years of practice). However, the use of online recruitment through social media constitutes a non‐probability convenience sampling approach, limiting the representativeness of the sample and, consequently, the generalizability of the results. A methodological limitation of this study is the reliance on DSM‐IV‐TR criteria rather than DSM‐5‐TR. Although DSM‐5‐TR is the current standard, the study site continued to use DSM‐IV‐TR at the time of data collection, and the available assessment tools had not yet been transitioned or revalidated for DSM‐5‐TR. However, the conceptual overlap and documented diagnostic continuity between DSM‐IV‐TR and DSM‐5‐TR reduce the likelihood of meaningful misclassification. The core symptom constructs explored in this study remain largely consistent across editions, suggesting limited impact on the interpretation of the findings. Future research would benefit from applying DSM‐5‐TR–aligned tools once they are fully adopted and validated in the local context. This study is subject to sampling bias due to online convenience sampling, which likely overrepresented digitally active, urban nurses and underrepresented rural or less connected groups. Voluntary participation may have attracted nurses with greater interest or confidence in ASD and excluded those with less than one year of experience, limiting input from newly trained graduates. These factors reduce the sample's representativeness and limit the generalizability of the findings to all nurses in Saudi Arabia. Self‐report measures can also introduce social desirability bias; nurses may overstate their knowledge or align responses with perceived best practices, particularly on sensitive topics such as intervention preferences. Future research should consider triangulating survey data with objective assessments such as case vignettes and observed clinical simulations to provide a more accurate picture. The cross‐sectional design limits causal inference; moreover, the geographic concentration of the sample may not reflect the diversity of knowledge and attitudes in remote or specialized regions. Furthermore, if those with more interest in or confidence in ASD are more likely to respond, participation bias may exist.

## 5 Conclusion

This study offers important empirical insights into Saudi nurses’ knowledge and clinical practices regarding ASD, revealing notable strengths in recognizing core diagnostic features alongside persistent gaps in understanding early onset criteria, the lifelong neurodevelopmental nature of ASD, and associated psychiatric comorbidities. The urgent need for comprehensive and culturally informed educational reforms is underscored by the misconceptions and systemic barriers that delay identification and intervention. Integrating targeted ASD‐specific curricula, ongoing professional development, and community engagement strategies in line with Saudi Vision 2030 can enhance early detection, reduce stigma, and facilitate multidisciplinary care pathways, according to these findings, which also emphasize the pivotal role that nurses play as frontline providers of autism care. It is essential for future research to use rigorous, multisite, and mixed‐method approaches to evaluate these interventions and explore the contextual barriers across diverse healthcare settings in Saudi Arabia.

## Author Contributions

Conceptualization: Monirah Albloushi Methodology: Monirah Albloushi, Reem Saeed Alghamdi. Investigation: Monirah Albloushi, Mona Alqahtani. Data curation: Mona Alqahtani. Formal analysis: Reem Saeed Alghamdi. Writing – original draft: Mona Alqahtani. Writing – review and editing: Monirah Albloushi, Reem Saeed Alghamdi. Supervision: Monirah Albloushi, Reem Saeed Alghamdi.

## Funding

The authors disclosed receipt of the following financial support for the research, authorship, and/or publication of this article: This study was funded by the Ongoing Research Funding Program (number: ORF‐2025‐1016) of King Saud University, Riyadh, Saudi Arabia.

## Conflicts of Interest

The authors report there are no competing interests to declare.

## Ethics Statement

Ethical approval was obtained from the Institutional Review Board of King Saud University (Approval No. 22–209).

## Data Availability

Data will be made available upon request.
